# Effects of water and fertilizer management on soil bacterial communities, enzyme activities, and nutrient availability in greenhouse tomatoes

**DOI:** 10.1371/journal.pone.0328793

**Published:** 2025-08-19

**Authors:** Xiaona Lyu, Hasnuri Mat Hassan, Yaling Zan, Jiongrui Tan

**Affiliations:** 1 Shanxi Technology Innovation Center of High Value-Added Echelon Utilization of Premium Agro-Products, Department of Life Sciences, Yuncheng University, Yuncheng, China; 2 School of Biological Sciences, Universiti Sains Malaysia, Penang, Malaysia; Universidade de Coimbra, PORTUGAL

## Abstract

Irrigation and fertilization strategies have been extensively employed to enhance the growth and yield of greenhouse tomatoes. However, the impacts of divergent fertilizer application patterns on soil microbial communities under water-saving irrigation regimes in China’s arid and semi-arid zones remain underexplored. In this study, a pot experiment was conducted in the greenhouse of Yuncheng University, Shanxi Province, incorporating three irrigation levels (I1, 90%–100% field capacity [Fs]; I2, 72%–80% Fs; I3, 54%–60% Fs) and four fertilization modes (C1, soluble organic–inorganic fertilizer combination; C2, sole soluble inorganic fertilizer; C3, sheep manure–inorganic fertilizer combination; C4, sole soluble organic fertilizer) to evaluate the effects of water-fertilizer management on the growth and physiological attributes of greenhouse tomatoes. Results demonstrated that irrigation regimes and fertilization patterns significantly modulated bacterial richness and diversity, as quantified by amplicon sequence variants (ASVs). The C1 treatment (soluble organic–inorganic fertilizer integration) exhibited the highest bacterial alpha diversity (Shannon index: 7.29). In beta diversity analysis, it induced the most distinct community structures, particularly under I3 (PCo1 = 17.96%) where it strongly diverged from C3. Microbial communities under I2 (PCo1 = 11.13%) showed greater homogeneity while preserving treatment-specific patterns, suggesting slight deficit irrigation balances stability and functional differentiation. The C1 treatment also elicited the most pronounced enhancement in soil enzyme activities, particularly phosphatase (PHO, 9.51 mg g ⁻ ¹) and catalase (CAT, 2.29 mL g ⁻ ¹). Conversely, reduced irrigation (I3) corresponded with decreased bacterial diversity, whereas slight deficit irrigation (I2) sustained higher microbial abundance compared to severe deficit irrigation. Additionally, I2 elevated soil pH (8.04), available phosphorus (AP: 10.39 mg kg ⁻ ¹), and soil nitrate nitrogen (SNO₃ ⁻ -N: 5.02 mg kg ⁻ ¹). These findings provide critical insights into optimizing water-fertilizer strategies to enhance microbial activity and nutrient cycling in greenhouse tomato production systems. Phylogenetic analysis identified Actinobacteriota (26.06%), Proteobacteria (25.89%), Chloroflexi (12.42%), and Acidobacteriota (11.03%) as the dominant bacterial phyla. Significant positive correlations were observed between invertase, urease, catalase, and alkaline phosphatase activities and microbial diversity indices (ASVs, ACE, Chao1, Shannon index). This study advances our understanding of how rhizosphere bacterial communities adapted to fertilization regimes under water stress, offering novel perspectives for precision management of greenhouse agroecosystems in water-constrained regions.

## 1. Introduction

Over recent decades, greenhouse cultivation has assumed an increasingly central role in global agricultural production systems [1]. As a high-value crop with substantial economic returns, tomato (*Solanum lycopersicum* L.) stands as one of the most widely cultivated vegetables in greenhouse systems worldwide. In China, it occupies the largest planting area among protected-cultivation vegetables [2]. However, tomatoes exhibit pronounced sensitivity to water and nutrient supply, with stringent requirements for both [3,4]. While consistent irrigation is essential, tomatoes also depend on well-drained soils to avoid root hypoxia and associated physiological disorders [5]. Data from the Ministry of Water Resources of China [6] show that total agricultural water consumption reached 368.23 billion m^3^, with less than 60% of irrigation water efficiently utilized by crops [7]. Excessive irrigation not only exacerbates water waste but also induces root anoxia, soil nutrient leaching, elevated greenhouse humidity, and intensified pest-disease pressures, collectively compromising vegetable yield and quality [8]. Emerging research indicates that moderate deficit irrigation—defined as applying water below full crop demand while maintaining optimal growth—maximizes crop water productivity, reduces agricultural water use [9], and mitigates water stress in arid/semi-arid regions [10,11]. To pursue higher yields and economic gains, farmers often over-rely on chemical fertilizers. China’s annual production and consumption of chemical fertilizers exceed 60 million tons, accounting for over one-third of global totals [12]. Nitrogen application rates frequently reach 2,000–4,000 kg N/hm^2^, far exceeding crop demand [13]. This practice not only promotes nitrate accumulation and reduces nitrogen use efficiency (with 30%–50% loss via leaching, volatilization, and runoff [14,15], but also elevates soil and crop nitrate levels, degrading fruit quality [16]. Consequently, there is a critical need for integrated water-fertilizer management strategies that balance yield optimization with minimized nutrient losses, such as precision irrigation scheduling, adoption of slow/controlled-release fertilizers, and integration of organic and inorganic fertilizers to enhance nutrient uptake efficiency [17].

Despite extensive research on irrigation and fertilization as independent factors, their interactive effects on soil microbial communities—particularly under water-scarce conditions—remain poorly understood. Soil microorganisms serve as key indicators of soil health, with microbial communities and enzymes playing indispensable roles in energy flow, nutrient cycling, and organic matter decomposition [18], all of which are fundamental to sustaining soil fertility [19,20]. Irrigation and fertilization influence soil bacterial communities by altering physicochemical properties and enzyme activities. For example, Qu et al. demonstrated that water deficit directly reduces bacterial abundance [8], highlighting water availability as a key driver of microbial community structure. Fertilization also profoundly impacts bacterial diversity: sole use of inorganic fertilizers has been linked to distinct bacterial and archaeal community compositions [21], while mineral nitrogen addition reduces microbial diversity and induces compositional shifts [22,23]. Conversely, Zhao et al. showed that pig dung-based organic-inorganic compound fertilizers enhance soil nutrient availability [24], microbial biomass, enzymatic activities, and nitrogen cycling processes while moderately increasing crop yields. Collectively, irrigation and fertilization shape soil microbial dynamics while regulating crop growth, development, and yield [25]. However, most existing studies focus on fertilizer application rates rather than fertilization patterns under water-scarce irrigation, creating a critical research gap in integrated water-fertilizer management for microbial community optimization.

This study employed high-throughput sequencing to investigate variability in microbial communities under different irrigation levels and fertilization patterns in a greenhouse tomato pot experiment. Specific objectives included: (1) Characterizing soil bacterial communities in greenhouse tomatoes via high-throughput sequencing; (2) Examining soil bacterial responses to divergent irrigation levels and fertilization patterns; (3) Assessing effects on soil enzymes and physicochemical properties; (4) Elucidating mechanisms through which irrigation-fertilization interactions influence microbial communities. Based on literature and study objectives, the following hypotheses were formulated:

**H1:** A soluble organic-inorganic fertilizer combination (C1) will significantly enhance soil enzyme activities and microbial diversity compared to other fertilization patterns.

**H2:** Slight deficit irrigation (I2) will promote higher soil bacterial richness and diversity than conventional irrigation (I1) or moderate deficit irrigation (I3), due to optimal moisture conditions for microbial activity.

**H3:** Irrigation levels and fertilization patterns will exhibit significant interactions affecting soil bacterial community composition and enzyme activities.

**H4:** Fertilization patterns incorporating organic inputs (C1, C3, C4) will more positively influence soil physicochemical properties and nutrient availability—particularly under I2—than soluble inorganic fertilization (C2).

## 2. Materials and methods

### 2.1. Experimental site description

A greenhouse tomato cultivation experiment was conducted from November 2022 to May 2023 at Yuncheng University’s Key Laboratory of Arid Agricultural Soil and Water Engineering (110°00’ E, 35°06’ N; 372 m elevation). The site features a warm temperate continental monsoon climate with mean annual values of 13.6°C temperature, 526.5 mm precipitation, and 2120.5 mm evaporation. The 666.67 m^2^ (25 m × 26.67 m) greenhouse utilized loamy soil with clay-loam texture. Soil physicochemical properties, measured before autumn 2022 transplanting, are shown in Table 1.

**Table 1:** Physical and chemical characteristics of tested soil in autumn 2022.

Soil Parameter	Values	Soil Parameter	Values
Total Nitrogen	0.94 ± 0.03 g kg^-1^	Bulk Density	0.82 ± 0.01g cm^-3^
Total Phosphorus	0.74 ± 0.01 g kg^-1^	pH	8.05 ± 0.01
Total Potassium	20.23 ± 1.23 g kg^-1^	Organic Matter	16.10 ± 1.21 g kg^-1^
Available Nitrogen	88.72 ± 3.25 mg kg^-1^	Field Capacity (*θ*_f_)	26.85% ± 1.2%
Available Phosphorus	18.11 ± 1.63 mg kg^-1^	Wilting Coefficient ( *θ*_w_)	8.4% ± 0.02%
Available Potassium	117.22 ± 10.56 mg kg^-1^	Electrical conductivity	127.14 ± 11.11μS cm^-1^

**Fig 1:**
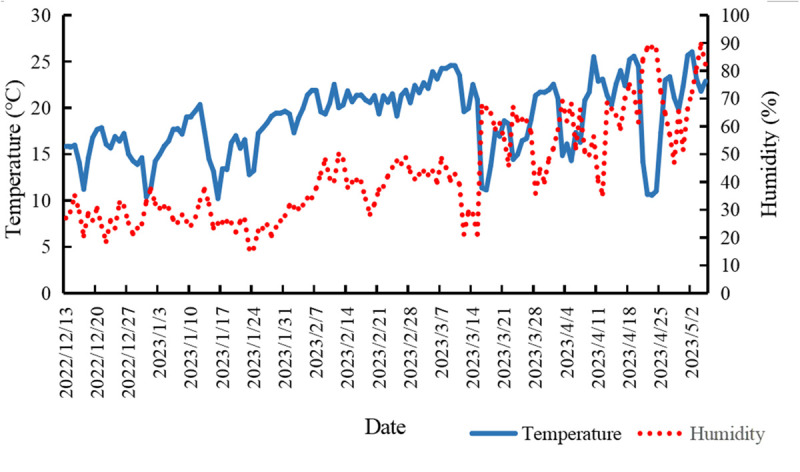
Average temperature during the growing season in the greenhouse.

During the experiment, a GPRS temperature and humidity recorder (Penghe Cold Chain Technology Co., Ltd.) was positioned at the greenhouse center to automatically log air temperature and humidity data. Fig 1 illustrates the mean temperature profile within the greenhouse across the entire growing season.

### 2.2. Experimental design

The experiment involved the manipulation of two primary factors: irrigation and fertilizer application patterns. Regarding irrigation, three different water levels were set, including conventional irrigation (I1, maintaining 90%−100% field water capacity), slight deficit irrigation (I2, maintaining 72%−80% field water capacity), and moderate deficit irrigation (I3, maintaining 54%−60% field water capacity). These irrigation levels were selected based on typical water usage patterns in commercial greenhouse tomato production in arid and semi-arid regions of China, where water scarcity is a pressing issue. Conventional irrigation (I1) represents the standard practice used by most growers to ensure optimal tomato growth, slight deficit irrigation (I2) aligns with water-saving strategies aimed at reducing water usage without compromising yield, and moderate deficit irrigation (I3) explores the lower limits of water application to assess the resilience of microbial communities and plant health under more severe water restrictions. Four patterns of fertilization were chosen: C1, application of both chemical and soluble organic fertilizer; C2, sole application of soluble chemical fertilizers; C3, combination of sheep manure and chemical fertilizer; and C4, sole application of soluble organic fertilizers. Conventional irrigation (I1) was applied to the control group (CK) without any fertilizer application. All fertilization modalities contained N 0.2, P₂O₅ 0.10, and K₂O 0.50 g kg ⁻ ¹. The CK received conventional irrigation (I1) but no fertilizers, serving as a baseline to assess the impact of irrigation alone without nutrient confounding effects. This design allows isolation of irrigation effects on soil microbial communities and plant performance by comparing against nutrient-amended treatments. The fertilizers used included potassium dihydrogen phosphate (51% P₂O₅, 34% K₂O), potassium sulfate (52% K₂O), and urea (46% N). Soluble organic fertilizers contained (% of dry matter) 15% nitrogen, 15% phosphorus and 30% potassium and 15% nitrogen, 5% phosphorus and 40% potassium. Additionally, sheep manure (1.50% N, 5.00% P, 4.00% K) served as the organic amendment [26]. The selected irrigation levels and fertilization patterns were designed to simulate realistic conditions encountered in commercial greenhouse tomato production, particularly in regions facing water scarcity and nutrient management challenges. By varying irrigation and fertilizer sources (organic, chemical, or their combination), the experiment systematically evaluates their interactions with water availability to influence soil properties, microbial activity, nutrient cycling, and plant performance. This setup ensures findings are directly applicable to optimizing water-fertilizer management strategies in greenhouse systems, thereby advancing sustainable agricultural practices under real-world constraints.

**Fig 2:**
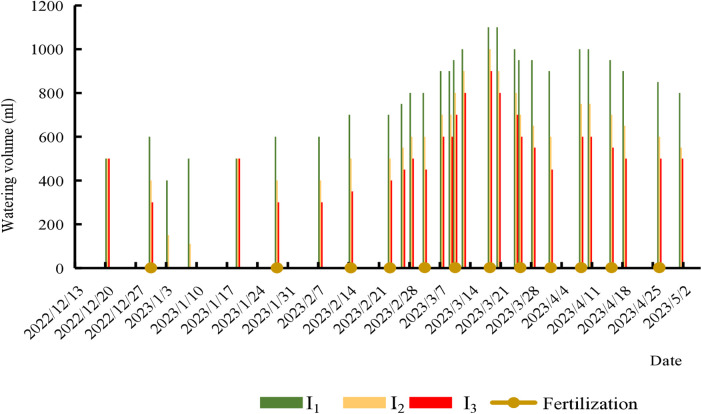
Irrigation and fertilization timing during the tomato growth stage.

The experiment utilized PVC pots (40 kg soil capacity, 40 cm row spacing) planted with the “Gao Rui Bright Pink” tomato cultivar (Weifang Pingyou Agricultural Technology Co., Ltd.), a high-yielding commercial variety selected for its color, market adaptability, and responsiveness to irrigation/nutrient management. At the 4-leaf stage, seedlings were transplanted (one plant per pot, 10 plants per treatment) with initial soil moisture restored to field capacity. Irrigation resumed after 7 days according to treatment protocols, maintaining target volumetric moisture levels via FDS-100 sensor monitoring: I1 (29.53%–32.7% v/v), I2 (23.6%–26.2% v/v), and I3 (17.68%–19.64% v/v). Plants were trellised at 30–40 cm height and topped after three fruit clusters developed. All non-experimental management followed standard greenhouse practices. Irrigation/fertilization schedules were recorded (Fig 2), with fruits harvested at full red maturity.

For each treatment, three replicate pots were harvested, and soil sampling was conducted at the root zone without damaging the root system. In each pot, soil cores were collected from three distinct points using a soil auger and combined to form a composite sample. Soil samples were processed by sieving (2 mm mesh) and dividing into three subsamples. One subsample was immediately stored in a liquid nitrogen tank and frozen at −80°C for subsequent microbial community analysis. A second subsample was maintained at 4°C for enzyme activity and soil nitrate (SNO₃ ⁻ -N) measurements. The final subsample was air-dried at room temperature, re-sieved, and used for physicochemical property analyses.

### 2.3. Soil physicochemical analyses

Soil pH was measured using a pH meter (Spectrum1 Technologies Inc., Aurora, IL, USA) at a soil-to-water ratio of 1:5 [27,28]. Soil nitrate nitrogen (SNO₃ ⁻ -N) was extracted with a 2 mol L ⁻ ¹ KCl solution and quantified using a flow analyzer (Auto Analyzer-Ⅲ, Germany). Soil available phosphorus (AP) was extracted with 0.5 mol L ⁻ ¹ NaHCO₃ and determined via the molybdenum blue colorimetric method. Soil available potassium (AK) was measured by flame photometry following leaching with 1.0 mol L ⁻ ¹ ammonium acetate (pH 7.0). Soil organic carbon (SOC) was determined using the potassium dichromate oxidation method (external heating at 170–180°C for 5 min), followed by titration with ferrous sulfate [29].

### 2.4. Soil enzyme activities assay

Four soil enzymes were assayed for activity: invertase (INV), urease (URE), catalase (CAT), and alkaline phosphatase (PHO). Soil invertase activity was determined using a colorimetric method with 3,5-dinitrosalicylic acid [30]. Urease activity was measured via the phenol-sodium hypochlorite colorimetric technique [31]. Catalase activity was quantified by titration with K₂MnO₄ [32]. Alkaline phosphatase activity was assessed using the nitrophenol colorimetric method [33].

### 2.5. Determination of soil microorganism samples

#### 2.5.1. DNA preparation, PCR amplification, and high-throughput sequencing.

Genomic DNA was extracted from each soil sample using the FastDNA® Spin Kit for Soil (MP Biomedicals, California, USA). DNA concentrations were quantified using a NanoDrop 2000 spectrophotometer (Thermo Fisher Scientific, Wilmington, DE, USA). Bacterial rRNA genes were amplified using the extracted DNA as templates. The V3-V4 hypervariable region of the 16S rRNA gene was amplified with primer sets 338F (5′-ACTCCTACGGGAGGCAGCAG-3′) and 806R (5′-GGACTACH VGGG T WTCTAAT-3′). PCR reactions (20 µL) comprised: 4 µL 5 × FastPfu buffer, 0.8 µL each primer (5 µM), 0.4 µL FastPfu DNA Polymerase, 0.2 µL BSA, 10 ng template DNA, and nuclease-free water. Thermal cycling involved: 95°C for 3 min; 30 cycles of 95°C (30 s), 55°C (30 s), 72°C (45 s); and final extension at 72°C for 10 min.

#### 2.5.2 Processing of illumina sequencing data.

Sequencing data processing: Bacterial PCR products were processed by Illumina Majorbio Biomedical Technologies (Shanghai, China), aliquoted, and sequenced (2 × 300 bp) on the Illumina MiSeq PE300 platform (Illumina, San Diego, CA, USA) [34]. Quality control was performed using fastp software [35] (https://github.com/OpenGene/fastp, v0.20.0) and FLASH [36] (http://www.cbcb.umd.edu/software/flash, v1.2.7), with the DADA2 plugin implemented in the QIIME 2 pipeline using default parameters [37]. Amplicon sequence variants (ASVs) were generated via DADA2 noise reduction, offering higher taxonomic resolution and reproducibility than OTU-based approaches [38]. To minimize the impact of sequencing depth on subsequent α- and β-diversity analyses, all samples were rarefied to 32,174 sequences (S1 Table). Even after rarefaction, the average Good’s coverage per sample remained at 99.65% (S2 Table). The final dataset comprised 1,254,786 sequences representing 55,870 ASVs (S3 Table). Taxonomic analysis of ASVs was conducted in QIIME 2 using the Naive Bayes classifier (or Vsearch/Blast) trained on the SILVA 16S rRNA gene database (v138).

#### 2.5.3 Alpha and beta diversity analysis.

The bacterial diversity of each sample was analyzed using an ASV (Amplicon Sequence Variant)-based approach. Alpha diversity indices (ASV richness and Shannon diversity) were calculated from raw counts using the estimate_richness function in the phyloseq R package and visualized with ggplot2 to assess biodiversity and species richness (alpha diversity) across irrigation and fertilization treatments for each sample [39]. Beta diversity analysis was performed on the full dataset to generate community structure comparison indices. Beta diversity at the ASV genotype level was quantified using unweighted UniFrac distances and visualized via Principal Coordinate Analysis (PCoA). The unweighted UniFrac distance matrix was processed and analyzed using R 3.3.1 and FastTree 2.1.3. Results revealed evolutionary relationships among diverse microbial assemblages and their relative abundances in the respective samples.

### 2.6. Statistical analyses

All statistical analyses were performed using SPSS version 17.0 (IBM Corp., Armonk, NY, USA) and R software (version 3.6.3). Descriptive statistics (means ± SDs)were computed for all soil physicochemical properties and enzyme activities. Analysis of variance (ANOVA) was conducted to evaluate the significant effects of irrigation levels and fertilization patterns on the measured parameters. Post hoc comparisons were carried out using Tukey’s Honest Significant Difference (HSD) test to identify specific differences between treatment groups. Bacterial alpha diversity indices (ASVs, ACE, Chao1, Shannon, and Simpson) were analyzed using the phyloseq package in R and visualized with ggplot2. Correlation analyses between soil enzymes, physicochemical properties, and bacterial diversity indices were performed using Pearson’s correlation coefficients and visualized with the corrplot package. Principal Coordinate Analysis (PCoA) based on unweighted UniFrac distances was conducted using the vegan package to assess beta diversity. Redundancy Analysis (RDA) was performed with the vegan package to explore associations between environmental factors and bacterial community composition. All statistical tests were two-tailed, and a significance level of p < 0.05 was applied for all analyses.

### 2.7 Experimental limitations

A key limitation of this study is the use of PVC pots rather than field-based experimentation. While pot trials enable controlled environmental conditions and precise manipulation of variables such as irrigation and fertilization, they cannot fully recapitulate the complexity of field ecosystems. The restricted soil volume in pots may constrain root growth and modify natural soil processes, potentially biasing results related to soil microbial activity and nutrient cycling. Additionally, pots are prone to edge effects, where root proximity to pot walls can alter root-soil interactions compared to open-field conditions, influencing water distribution, root architecture, and the broader microbial microenvironment. Another constraint is the limited soil volume, which may hinder the full expression of plant responses to water and nutrient stress. In natural field settings, plants typically access larger soil volumes, supporting more extensive root systems and potentially distinct interactions with soil microbiomes. Future research should incorporate field experiments to validate these findings under more realistic conditions and to investigate how these interactions scale in environments where factors like temperature variability, wind, and soil heterogeneity are operational. Despite these limitations, the pot-based approach provides valuable controlled-condition insights, enabling isolation of specific factors and their direct impacts on plant growth, soil properties, and microbial dynamics.

## 3. Results

### 3.1 Soil physico-chemical properties and enzyme activities

Table 2 demonstrates that irrigation and fertilization significantly influence soil physicochemical properties and enzyme activities. The activities of phosphatase (PHO), catalase (CAT), urease (URE), and invertase (INV) decreased with reduced irrigation levels in the order I1 > I2 > I3. Specifically, CAT activity reached a maximum of 2.29 mL g ⁻ ¹ soil under I1 (conventional irrigation) with the C1 treatment, but significantly decreased to 1.08 mL g ⁻ ¹ under I2 (slight deficit irrigation) and 0.96 mL g ⁻ ¹ under I3 (moderate deficit irrigation). Soil pH values increased as irrigation decreased, ranging from 7.90 under I1 (C1) to 8.89 under I3 (C1). Available phosphorus (AP) also varied across treatments, with AP under I3 (C1) reaching 11.29 mg kg ⁻ ¹ compared to 8.83 mg kg ⁻ ¹ under I1 (C1). Soil nitrate nitrogen (SNO₃ ⁻ -N) content peaked under I2 (C1) at 5.57 mg kg ⁻ ¹ and slightly decreased to 5.52 mg kg ⁻ ¹ under I3 (C1). Among all treatments, the C2 (soluble chemical fertilizer only) treatment consistently exhibited lower enzyme activities across all parameters, while the C1 treatment (organic-inorganic fertilizer combination) showed higher phosphatase (PHO) and urease (URE) activities than other fertilization patterns. The highest recorded activities for INV, CAT, URE, and PHO were 2.80 mL g ⁻ ¹, 2.29 mL g ⁻ ¹, 157.12 mg NH₃-N g ⁻ ¹ d ⁻ ¹, and 9.51 mg g ⁻ ¹, respectively.

**Table 2:** Effect of different water and fertilizer application patterns on soil enzymes and physico-chemical properties of greenhouse tomatoes.

Irrigation Level	Fertilization Level	pH	SOC (g kg^-1^)	PHO (mg g^-1^)	CAT (mL g^-1^)	URE (mg NH_3_-N g^-1^ d^-1^)	INV (mL g^-1^)	SNO_3_^—^N (mg kg^-1^)	AP (mg kg^-1^)	AK (mg kg^-1^)
I1	C1	7.90 d	11.58 ab	9.51 a	2.29 a	157.12 a	2.80 a	3.33 e	8.83 f	98.96 e
	C2	8.20 bc	10.05 e	2.66 g	1.24 c	67.95 g	1.00 d	4.80 b	12.61 b	121.20 a
	C3	7.90 d	11.46 bc	7.28 d	1.31 c	78.42 f	1.85 bc	4.09 d	8.49 f	105.90 c
	C4	7.98 d	11.21 c	9.46 ab	1.65 b	113.63 c	2.05 b	4.11 d	9.17 e	105.40 c
	CK	7.89 d	10.21e	9.25 b	1.56 bc	121.00 b	2.15b	2.93 f	11.35 cd	88.57 h
I2	C1	8.26 b	9.34 f	2.22 h	1.08 e	57.95 h	0.90d	5.57 a	13.47 ab	103.90 cd
	C2	8.03 cd	10.09e	3.54 f	1.25 c	43.12 i	0.65 e	4.88 b	7.68 g	99.32 e
	C3	8.05 cd	9.53f	8.34 c	1.45 bc	81.62 e	1.60 c	4.91 b	11.72 c	90.72 g
	C4	7.82 e	11.72a	9.21 b	1.44 bc	98.47 d	1.85bc	4.71 c	8.69 f	90.41 g
I3	C1	8.89 a	9.23g	2.49 g	0.96 f	53.4 h	0.70 e	5.52 a	11.29 cd	106.60 b
	C2	8.05 cd	11.48 bc	7.24 d	1.14 d	60.71 g	1.25 cd	4.84 b	7.79 g	102.50 d
	C3	8.08 c	10.77 de	5.55 e	1.08 e	75.70 f	1.51 c	5.36 ab	10.22 d	92.43 f
	C4	8.12 bc	10.85 d	5.69 e	1.12 d	46.4 i	0.55f	2.57 g	13.83 a	94.18 ef

Notes: I1, conventional irrigation (90%–100% Fs); I2, slight deficit irrigation (72%–80% Fs). I3, moderate deficit irrigation (54%–60% Fs). Fs is the field’s water storage capacity. C1, soluble organic fertilizer and chemical fertilizer; C2, soluble chemical fertilizer only; C3, sheep manure and chemical fertilizer; and C4, soluble organic fertilizer only. CK is a control with no fertilizer. pH, soil pH value. SOC, soil organic carbon; PHO, alkaline phosphatase; CAT, catalase; URE, urease; and INV, invertase. SNO_3_^−^-N, Soil nitrate nitrogen; AP, available phosphorus; and AK, available potassium. Different letters after the same column of numbers indicate significant differences (p < 0.05).

**Fig 3:**
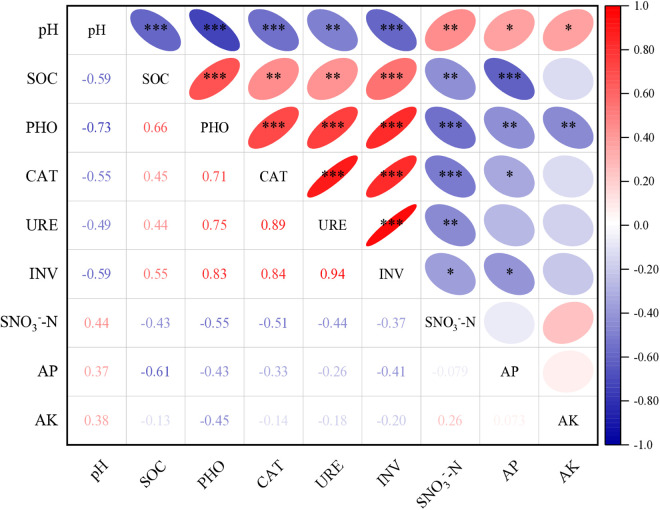
Pearson’s correlation analysis for soil enzymes and physico-chemical properties. pH, soil pH value; SOC, soil organic carbon; PHO, alkaline phosphatase; CAT, catalase; URE, urease; and INV, invertase. SNO_3_^−^-N, Soil nitrate nitrogen; AP, available phosphorus; and AK, available potassium. The gradient of the legend reflects the degree of the correlation, whereas the slope of the ellipse shows a negative or positive correlation. The shape of the ellipse represents the strength of the link. Asterisks ‘*’, ‘**’, and ‘***’ indicate significance at the 0.05, 0.01, and 0.001 probability levels, respectively.

Correlation analysis revealed that soil organic carbon (SOC) and soil enzyme activities were highly positively correlated (p < 0.01), while both were significantly negatively correlated with soil pH (p < 0.05) and SNO₃ ⁻ -N (p < 0.01) (Fig 3). Additionally, a positive correlation was observed between soil pH and SNO₃ ⁻ -N, whereas significant positive correlations were found among INV, URE, CAT, and PHO activities. Soil pH exhibited significant positive relationships with available potassium (AK), available phosphorus (AP), and soil nitrate nitrogen (SNO₃ ⁻ -N). These results further indicate that irrigation and fertilization patterns significantly impact soil physicochemical properties.

### 3.2 Soil sample sequencing results and venn diagram

**Fig 4:**
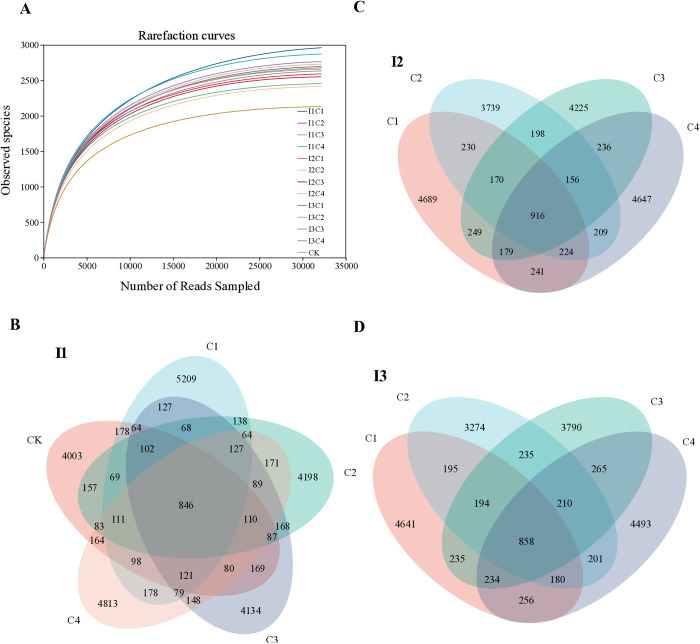
Soil bacterial rarefaction curve (A) and venn diagram (B, I1; C, I2; D, I3). I1, conventional irrigation (90%–100% Fs); I2, slight deficit irrigation (72%–80% Fs). I3, moderate deficit irrigation (54%–60% Fs). Fs is the field’s water storage capacity. C1, soluble organic fertilizer and chemical fertilizer; C2, soluble chemical fertilizer only; C3, sheep manure and chemical fertilizer; and C4, soluble organic fertilizer only. CK is a control with no fertilizer.

The rarefaction curve (Fig 4A) indicates that the sequencing depth was sufficient to capture the core characteristics of microbial communities in soil samples, showing a rapid increase in sample coverage with read counts, followed by a plateau. The number of unique bacterial ASVs followed the pattern C1 > C4 > C3 > C2 > CK under full irrigation (I1) (Fig 4B) and C1 > C4 > C3 > C2 under deficit irrigation (I2 and I3) (Figs 4C, 4D). Additionally, the quantity of distinct bacterial ASVs decreased with reduced irrigation levels. The richness and diversity of greenhouse tomato bacterial communities varied significantly across water-fertilizer treatments. Diversity followed the trend C1 > C4 > C3 > C2, while richness exhibited C4 > CK > C1 > C3 > C2 under I1 and C1 > C4 > C3 > C2 under I2 and I3. The C1 treatment (soluble organic-chemical fertilizer combination) with slight deficit irrigation (I2) significantly enhanced soil bacterial community richness and diversity.

### 3.3 Bacterial alpha and beta diversity

Table 3 illustrates the variation in soil microbial communities across different water-fertilizer management systems in greenhouse tomatoes. ASVs represent observed species, while bacterial alpha diversity was estimated using ACE, Chao1 richness indices, and Shannon, Simpson, and Coverage diversity indices. Irrigation significantly influenced ASVs, ACE, Chao1 (P < 0.001), Coverage (P < 0.01), and Shannon index (P < 0.05). Reduced irrigation levels were associated with decreases in ASVs, ACE, and Chao1 indices, though the Coverage index peaked at 99.72% under I3. Fertilization patterns significantly affected ASVs, ACE, Chao1 (P < 0.001), and Coverage (P < 0.05) but had no effect on the Shannon index (P > 0.05).

**Table 3:** Effect of different water and fertilizer application patterns on the richness and diversity of the bacteria community of greenhouse tomatoes.

Irrigation Level	Fertilization Level	ASVs	ACE	Chao1	Shannon	Simpson	Coverage (%)
I1	C1	2961 ± 113 a	3089.13 ± 137.22 a	3002.66 ± 121.12 a	7.21 ± 0.13 bc	0.0018 ± 0.0010 e	99.22 ± 0.13 g
	C2	2591 ± 19 f	2639.62 ± 39.71 e	2605.07 ± 26.81 e	7.19 ± 0.09 bc	0.0015 ± 0.0006 f	99.65 ± 0.19 d
	C3	2676 ± 5 d	2736.12 ± 23.15 d	2694.44 ± 9.62 d	7.18 ± 0.05 bc	0.0016 ± 0.0002 ef	99.57 ± 0.16 f
	C4	2872 ± 9 b	2936.34 ± 32.52 b	2887.76 ± 16.18 b	7.29 ± 0.04 a	0.0013 ± 0.0003 g	99.55 ± 0.13 f
I2	C1	2765 ± 9 c	2822.00 ± 17.27 c	2776.71 ± 10.87 c	7.23 ± 0.03 b	0.0013 ± 0.0001 g	99.60 ± 0.05 e
	C2	2415 ± 5 g	2443.83 ± 5.79 g	2419.52 ± 2.73 g	6.93 ± 0.17 d	0.0034 ± 0.0015 b	99.78 ± 0.07 c
	C3	2550 ± 16 f	2570.61 ± 22.60 f	2552.27 ± 16.32 f	7.07 ± 0.10 d	0.0024 ± 0.0008 d	99.83 ± 0.08 b
	C4	2729 ± 5 cd	2783.13 ± 44.89 d	2743.23 ± 15.26 cd	7.11 ± 0.19 c	0.0034 ± 0.0033 b	99.64 ± 0.28 d
I3	C1	2696 ± 8 d	2743.19 ± 12.90d	2705.84 ± 2.74 d	7.12 ± 0.17 c	0.0025 ± 0.0019 d	99.65 ± 0.14 d
	C2	2130 ± 25 h	2150.69 ± 235.74 h	2135.08 ± 245.54 h	6.59 ± 0.84 e	0.0101 ± 0.0148 a	99.84 ± 0.15 a
	C3	2454 ± 27 g	2483.74 ± 40.30 g	2461.14 ± 29.51 g	7.03 ± 0.23 de	0.0031 ± 0.0028 cd	99.77 ± 0.18 c
	C4	2634 ± 4 e	2687.22 ± 21.71 de	2647.83 ± 8.94 de	7.09 ± 0.10 d	0.0027 ± 0.0016 c	99.62 ± 0.16 e
	CK	2658 ± 5 d	2699.66 ± 14.65 de	2667.78 ± 6.31 de	7.20 ± 0.04 bc	0.0015 ± 0.0002 f	99.68 ± 0.10 cd
Irrigation		^***^	^***^	^***^	^*^	ns	^**^
Fertilization		^***^	^***^	^***^	ns	ns	^**^
Irrigation× Fertilization		ns	ns	ns	ns	ns	ns

Notes: I1, conventional irrigation (90%–100% Fs); I2, slight deficit irrigation (72%–80% Fs). I3, moderate deficit irrigation (54%–60% Fs). Fs represents field water holding capacity. C1, soluble organic fertilizer and chemical fertilizer; C2, soluble chemical fertilizer only; C3, sheep manure and chemical fertilizer; and C4, soluble organic fertilizer only. CK is a control with no fertilizer. Significant variations (p < 0.05) between soil samples are denoted by different letters within each column. At p > 0.05, ns (not significant).

* 0.01 < p ≤ 0.05;

**0.001 < p ≤ 0.01;

*** p ≤ 0.001 ASVs reflect observed species, while ACE and Chao1 represent bacterial community richness, and Shannon, Simpson, and Coverage represent bacterial community diversity.

Under I1, the order of ASVs, ACE, and Chao1 was C1 > C4 > CK > C3 > C2, whereas under I2 and I3, the order shifted to C1 > C4 > C3 > C2. The Shannon index followed a consistent pattern across all irrigation levels: CK > C1 > C4 > C3 > C2. C1 exhibited the lowest Coverage, while C3 showed the highest across irrigation levels. Neither irrigation nor fertilization patterns significantly affected the Simpson index, and no significant interaction was detected between them.

**Fig 5:**
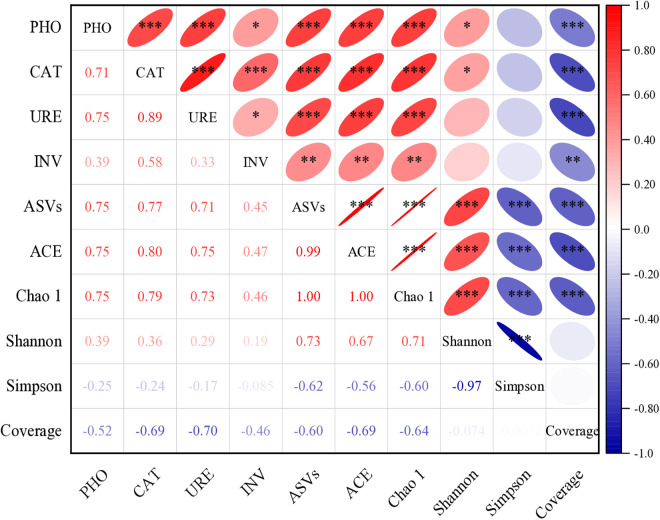
Pearson’s correlation analysis for soil enzymes and indices of bacterial alpha diversity. SOC, soil organic carbon; PHO, alkaline phosphatase; CAT, catalase; URE, urease; and INV, invertase. ASVs reflect observed species, while ACE and Chao1 represent bacterial community richness and Shannon, Simpson, and Coverage represent bacterial community diversity. *p < 0.05, **p < 0.01 and ***p < 0.001.

ASVs, ACE, and Chao1 were strongly positively correlated with phosphatase (PHO), catalase (CAT), urease (URE), and invertase (INV) activities (p < 0.001), while Shannon indices were positively correlated with PHO and CAT (p < 0.05). The Coverage index was strongly negatively correlated with all soil enzymes (p < 0.001) (Fig 5).

**Fig 6:**
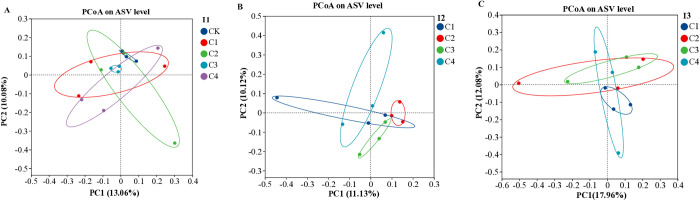
The Beta diversity of soil bacterial community under different water and fertilizer application patterns (A, I1; B, I2; C, I3). I1, conventional irrigation (90%–100% Fs); I2, slight deficit irrigation (72%–80% Fs). I3, moderate deficit irrigation (54%–60% Fs). Fs represents the field’s water storage capacity. C1, soluble organic fertilizer and chemical fertilizer; C2, soluble chemical fertilizer alone; C3, sheep manure and chemical fertilizer; and C4, soluble organic fertilizer only. CK is a control that receives no fertilizer.

Bacterial beta diversity was analyzed phylogenetically using unweighted UniFrac distances. Principal Coordinate Analysis (PCoA; Fig 6) visualized community structural variation, with the first two coordinates (PCo1 and PCo2) explaining distinct proportions of variance. For the three irrigation scenarios, under I1 (conventional irrigation, 90%–100% Fs), PCo1 and PCo2 accounted for 13.06% and 10.08% of the variance, respectively (Fig 6A); under I2 (slight deficit irrigation, 72%–80% Fs), PCo1 and PCo2 explained 11.13% and 10.12% of the variance (Fig 6B); and under I3 (moderate deficit irrigation, 54%–60% Fs), PCo1 and PCo2 accounted for 17.96% and 12.08% of the variance (Fig 6C). Microbial communities under I2 showed higher overall similarity than those under I1 and I3. Under I1, C3 differed significantly from CK, while C1, C2, C3, and C4 showed no significant separation (Fig 6A). Under I2, C2, C3, and C4 maintained identifiable community structures, with C2 and C3 showing stronger clustering (Fig 6B). Under I3, C1 and C3 were significantly divergent, while C2, C3, and C4 displayed closer relationships (Fig 6C).

### 3.4 Bacterial community composition and abundance

Table 4 shows that the dominant bacterial phyla with relative abundances exceeding 1% in greenhouse tomato soils were led by Actinobacteriota (26.06%), followed by Proteobacteria (25.89%), Chloroflexi (12.42%), and Acidobacteriota (11.03%), with lower abundances observed in other phyla. Water-fertilizer management significantly altered the structure of dominant bacterial communities: Actinobacteriota dominated across all treatments, particularly in the C1 treatment under all irrigation levels, though its abundance decreased with reduced irrigation in C2 and C3 treatments, exhibited a rise-then-fall pattern in C4 (peaking at 28.20% under I2). Proteobacteria showed the highest abundances in the C2 treatment under I1 and in the C1 treatment under I2 and I3. Slight deficit irrigation (I2) enhanced the abundance of Proteobacteria in C1 and C4 treatments (e.g., reaching 28.58% in C1), while C1 had significantly higher Chloroflexi (11.78%) and lower Actinobacteriota (8.03%) compared to C4. The top four phyla (Actinobacteriota, Proteobacteria, Chloroflexi, Acidobacteriota) remained dominant in all CK, C1-C4 treatments. Reduced irrigation increased the abundances of Chloroflexi and Acidobacteriota in C1 and C3 but decreased them in C2 and C4. Under conventional irrigation (I1), the C1 treatment had higher abundances of Actinobacteriota and Proteobacteria but lower Chloroflexi in soils. The results indicate that water-fertilizer management patterns can regulate soil microbial community structures, where the C1 treatment under slight deficit irrigation (I2) promotes the growth of beneficial phyla such as Proteobacteria, Actinobacteriota, and Chloroflexi, while regulating the abundance of Acidobacteriota in specific treatments.

**Table 4:** Effect of different water and fertilizer application patterns on soil bacterial phyla abundance in greenhouse tomatoes.

Irrigation Level	Fertilization Level	*Actinobacteriota*	*Proteobacteria*	*Chloroflexi*	*Acidobacteriota*	Cyanobacteria	Gemmatimonadota	Bacteroidota	Myxococcota	Firmicutes	unclassified_k__norank_d__Bacteria	Patescibacteria
I1	C1	28.23	27.35	10.74	8.84	4.35	4.52	3.53	3.09	3.13	2.18	0.83
	C2	27.26	27.51	12.67	12.78	0.94	4.47	3.65	2.78	1.70	1.49	0.94
	C3	25.74	25.80	13.50	10.82	4.39	3.90	4.26	3.50	1.68	1.44	1.37
	C4	25.50	27.47	13.17	11.50	1.67	4.45	4.53	3.15	1.42	1.96	1.04
	CK	26.33	26.27	13.13	11.36	3.44	3.98	4.08	2.89	2.02	1.42	1.01
I2	C1	28.43	28.58	11.78	8.03	3.21	4.62	3.70	3.34	1.66	1.91	1.17
	C2	23.39	23.61	13.33	12.53	10.10	3.51	3.71	2.22	1.47	1.25	0.95
	C3	25.08	24.92	12.21	12.55	5.78	4.44	3.24	2.86	2.30	1.54	1.46
	C4	28.20	27.39	11.49	8.97	4.47	3.86	3.54	3.03	3.01	1.85	0.76
I3	C1	29.79	25.01	11.75	8.44	6.23	4.34	3.42	3.04	1.89	1.90	0.93
	C2	21.62	24.31	12.42	12.15	13.88	3.90	3.10	2.16	0.98	1.23	0.96
	C3	21.98	23.78	13.49	15.62	6.47	4.37	3.63	2.81	1.37	1.35	1.02
	C4	27.22	24.60	11.82	9.84	8.20	4.14	3.11	3.07	1.98	1.54	1.01
Average		26.06	25.89	12.42	11.03	5.63	4.19	3.65	2.92	1.89	1.62	1.03

Notes: I1, conventional irrigation (90%–100% Fs); I2, slight deficit irrigation (72%–80% Fs). I3, moderate deficit irrigation (54%–60% Fs). Fs is the field water holding capacity. C1, soluble organic fertilizer and chemical fertilizer; C2, soluble chemical fertilizer only; C3, sheep manure and chemical fertilizer; C4, soluble organic fertilizer only. CK, a control with no fertilizer.

**Fig 7:**
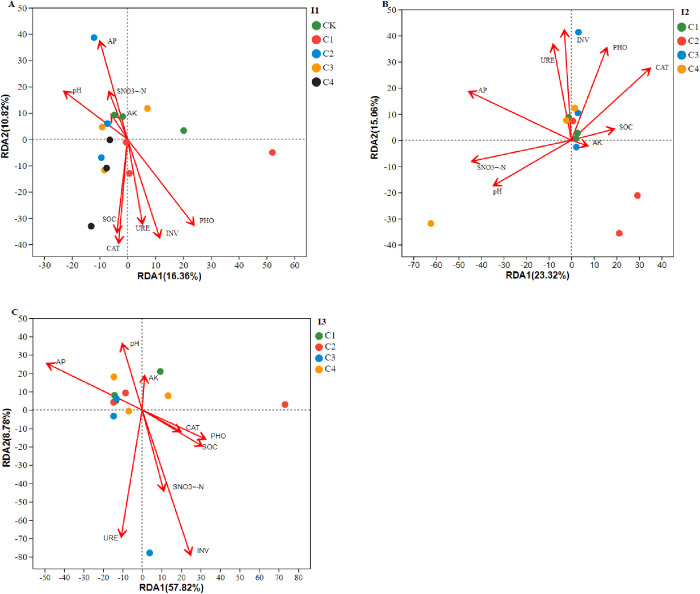
Redundancy analysis of soil environmental factors and bacterial dominant communities under different water and fertilizer application patterns of greenhouse tomatoes (A, I1; B, I2; C, I3). I1, conventional irrigation (90%–100% Fs); I2, slight deficit irrigation (72%–80% Fs). I3, moderate deficit irrigation (54%–60% Fs). Fs is the field’s water storage capacity. C1, soluble organic fertilizer and chemical fertilizer; C2, soluble chemical fertilizer only; C3, sheep manure and chemical fertilizer; and C4, soluble organic fertilizer only. CK is a control with no fertilizer. pH, soil pH value. SOC, soil organic carbon; PHO, alkaline phosphatase; CAT, catalase; URE, urease; and INV, invertase. SNO_3_^−^-N, Soil nitrate nitrogen; AP, available phosphorus; and AK, available potassium.

Redundancy Analysis (RDA) was employed to evaluate the relationships between environmental factors and bacterial community composition. The first two RDA axes explained 16.36% and 10.82% of the total variation under I1 (Fig 7A), 23.32% and 15.06% under I2 (Fig 7B), and 57.82% and 8.78% under I3 (Fig 7C). In I1 treatments (CK, C1, C4), soil enzymes and soil organic carbon (SOC) were strongly associated with bacterial community dispersion. Additionally, soil available phosphorus (AP) was linked to bacterial community distribution in C2 under I2, though C2 clustered on the opposite side of the AP axis and closer to the origin. Under I3, urease (URE) and invertase (INV) were correlated with bacterial community distribution in C3.

### 3.5 Relationship of the bacterial community with environmental factors

**Fig 8:**
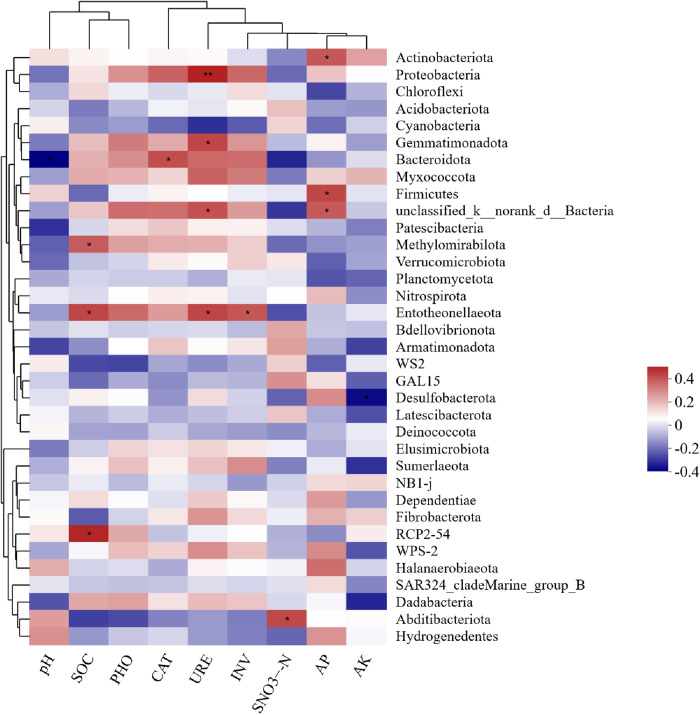
Correlation coefficients (R) between bacterial phyla and soil environmental factors under different water and fertilizer application patterns of greenhouse tomatoes. Correlation coefficients (R) between bacterial genera and soil characteristics across MP treatments. Red indicates a positive correlation, whereas blue indicates a negative correlation. Darker colors indicate a higher correlation. A single asterisk (*) denotes statistical significance at p < 0.05, whereas a double asterisk (**) indicates statistical significance at p < 0.01.

The correlation heatmap of bacterial phyla revealed that Bacteroidota was negatively associated with soil pH (p < 0.05). Soil organic carbon (SOC) exhibited positive correlations with RCP2–54 (p < 0.01), Entotheonellaeota (p < 0.05), and Methylomirabilota (p < 0.01). Soil urease (URE) was positively correlated with Proteobacteria, Gemmatimonadota, unclassified_k__norank_d__Bacteria, and Entotheonellaeota, while Bacteroidota showed a positive association with soil catalase (CAT). Additionally, Abditibacteriota and Entotheonellaeota were positively linked to soil nitrate nitrogen (SNO₃ ⁻ -N) and invertase (INV), respectively. Soil available phosphorus (AP) was strongly positively correlated with Actinobacteriota, Firmicutes, and unclassified_k__norank_d__Bacteria. Desulfobacterota exhibited a negative association with soil available potassium (AK) (Fig 8). The constructed correlation heatmap indicated that most bacterial phyla were associated with soil enzymes and physicochemical properties, with the exception of phosphatase (PHO).

## 4. Discussion

### 4.1 Soil enzymes affected by irrigation

Irrigation and fertilization practices are recognized as key drivers of soil nutrients, microbial communities, and enzymatic activities—critical components of agricultural ecosystems [40,41]. Moderate irrigation has been shown to enhance soil fertility and microbial function by minimizing loss of soil available nutrients and promoting nutrient uptake efficiency [42]. For instance, Muhammad et al. [41] reported higher nitrogen (N) and phosphorus (P) immobilization in microbial biomass under moderate water deficit conditions. In this study, soil nitrate nitrogen (SNO₃ ⁻ -N) peaked at I2 (slight deficit irrigation), while available phosphorus (AP) was highest at I3 (moderate deficit irrigation). These results align with Wu et al. [43], who observed reduced soil organic carbon (SOC) under moderate water stress, likely due to enhanced surface microbial activity and oxygenation under limited irrigation, which accelerates organic matter mineralization and decomposition. Our findings also resonate with Li et al. [44], who demonstrated that higher soil water content promotes microbial growth and metabolic enzyme activity, thereby enhancing nitrogen use efficiency.

### 4.2 Soil enzymes affected by fertilization

Under I1 (conventional irrigation), phosphatase (PHO), catalase (CAT), and urease (URE) activities followed the order C1 > C4 > CK > C3 > C2, while invertase (INV) followed C1 > CK > C4 > C3 > C2. The inclusion of liquid organic fertilizers in C1 (organic-inorganic combination) and C4 (soluble organic fertilizer) significantly elevated soil enzyme activities, attributable to increased organic matter availability as a substrate for microbial decomposition. This supports the production of enzymes like PHO, CAT, and URE, which mitigate oxidative stress in soil microbial communities by scavenging reactive oxygen species (ROS) generated during organic matter breakdown and nutrient cycling. This part verifies hypothesis H1. Conversely, the C2 treatment (soluble chemical fertilizer) exhibited suppressed enzyme activity, underscoring the indispensable role of organic matter in sustaining microbial enzymatic functions [45]. The strong positive correlation between soil humus content and enzyme activity further explains the superior performance of C1 and C4, where organic inputs enhanced microbial metabolism and nutrient cycling efficiency.

### 4.3 Soil enzymes affected by irrigation and fertilization

Pearson correlation analysis revealed significant positive relationships between soil enzymes (PHO, CAT, URE, and INV) and bacterial diversity indices (ASVs, ACE, Chao1) as well as richness (Shannon index) (p < 0.05), with the exception that URE and INV showed no significant correlation with richness. Irrigation influences enzyme activity predominantly by modulating soil moisture, a critical factor for microbial metabolism and extracellular enzyme production. Under full irrigation (I1), optimal soil moisture supports robust microbial growth, thereby enhancing enzyme activity. Conversely, slight deficit irrigation (I2) imposes mild water stress, which may stimulate microbial communities to optimize nutrient uptake under limited water availability, thereby upregulating enzymes involved in nutrient cycling (e.g., CAT, URE). This finding aligns with prior research demonstrating that moderate water deficits can enhance soil microbial activity and enzymatic processes. This part verifies hypothesis H3.

### 4.4 Soil microbes affected by irrigation

Soil water content is a primary driver of soil microbial community structure and activity, as it directly influences soil chemical processes, which in turn affect microbial diversity, ecosystem stability, and soil productivity [46]. However, our study revealed that bacterial alpha diversity and richness were influenced not only by soil moisture but also by the interaction between fertilization patterns and irrigation levels, underscoring the complex role of irrigation in shaping microbial activity (Table 3). This suggests that soil water content alone cannot fully explain the observed variations in microbial diversity. ASVs, ACE, and Chao1 indices demonstrated that C1 (soluble organic fertilizer combined with chemical fertilizer) exhibited significantly higher richness across all irrigation levels compared to other fertilization patterns (p < 0.05; Table 3). This is further supported by the trend in bacterial unique ASV counts (C1 > C4 > C3 > C2 under I2 and I3) and the overall richness order (C1 > C4 > C3 > C2) in Figs 4C, and 4D. Consistent with prior research, the Shannon index (p < 0.05) indicated that chemical fertilization (C2) reduced microbial diversity, while organic fertilizers (C1, C4) tended to enhance it [47]. A decline in both bacterial richness and diversity with decreasing irrigation levels aligned with findings from Sun et al. [48], who reported significant reductions in Chao1 and Shannon indices under lower irrigation regimes. These results corroborate the hypothesis that optimal soil moisture promotes bacterial diversity and community stability, whereas extreme dryness or wetness has detrimental effects [41]. The present findings are consistent with Li et al. [49], highlighting irrigation as a critical factor in regulating soil microbial community diversity.

### 4.5 Soil microbes affected by fertilization

Additionally, the C1 treatment exhibited significantly higher bacterial diversity compared to other fertilization patterns (Table 3). Results indicate that reducing chemical fertilizer inputs or combining them with organic fertilizers can enhance soil bacterial community alpha diversity in tomato crops. Partial substitution of nitrogen fertilizers with organic amendments may mitigate the negative impacts of conventional fertilizers on soil bacterial alpha diversity. As noted by Agba et al. [50], organic fertilizers provide soil bacteria and other microorganisms with labile carbon substrates, thereby promoting microbial diversity. This part verifies hypothesis H1. The enhanced microbial diversity under organic fertilization carries critical ecological significance: diverse microbial communities can improve soil resilience to environmental stresses and suppress plant pathogens through competitive exclusion.

### 4.6 Soil microbes affected by irrigation and fertilization

Based on unweighted UniFrac distances (Fig 6), both alpha and beta diversity exhibited significant positive associations with irrigation and fertilization patterns (Table 3). As irrigation levels decreased, the positive correlation between bacterial diversity and fertilization patterns strengthened, particularly under slight deficit irrigation (I2) (p < 0.05), indicating that reduced water availability amplified the impact of fertilization on bacterial community structure. The interaction between irrigation and fertilization suggests that irrigation influences not only soil moisture directly but also modulates fertilization efficacy by altering nutrient availability and microbial activity. This phenomenon may be attributed to enhanced nitrogen (N) accessibility for soil microorganisms under mild water deficit [51], further supported by the peak soil nitrate nitrogen (SNO₃ ⁻ -N) levels observed under I2 (Table 2). Under conventional irrigation (I1), the four fertilization treatments (notably C3) diverged significantly from the control (CK), highlighting the effect of fertilization on microbial diversity. Under deficit irrigation, sole chemical fertilizer (C2), sheep manure + chemical fertilizer (C3), and soluble organic fertilizer (C4) yielded distinct community structures, with C2 and C3 showing stronger clustering (Fig 6B). This aligns with Zhang et al. [52], who proposed that soil bacterial communities are highly sensitive to fertilization types, as evidenced by their rapid and divergent responses to different fertilization regimes. Moreover, organic-inorganic fertilizer combinations (C1) had a more profound effect on reshaping soil bacterial communities than single fertilizer inputs, supported by the Shannon index trend (CK > C1 > C4 > C3 > C2; Table 3).

### 4.7 Influence of irrigation and fertilization on bacterial composition

This study demonstrated that irrigation and fertilization patterns significantly influenced the bacterial community structure of greenhouse tomatoes (Table 4). Actinobacteriota, Proteobacteria, Chloroflexi, and Acidobacteriota were the dominant phyla across all three irrigation levels (Table 4), consistent with previous findings by Wu et al. [11]. Varying irrigation and fertilization regimes altered soil physicochemical properties, which are closely linked to soil bacterial communities. While both factors significantly impacted bacterial richness and diversity, no significant interaction was detected between irrigation and fertilization patterns in shaping bacterial community structure (Table 3). These results align with Muhammad et al. [41], who reported independent effects of irrigation and nitrogen fertilization on bacterial populations. This part verifies hypothesis H3.

### 4.8 Interaction between soil nutrients and microbial communities

Our findings revealed significant variations in bacterial community structure at the phylum level across irrigation and fertilization patterns (Table 4). Under slight deficit irrigation (I2), Proteobacteria, Acidobacteriota, and Chloroflexi exhibited markedly higher abundances in C1 and C4 soils. These phyla are integral to critical soil functions, including nitrogen fixation and organic matter decomposition. Their increased prevalence under specific treatments suggests a synergistic response to irrigation-induced moisture conditions and nutrient availability, directly influencing the microbial community’s functional potential. This study demonstrated that changes in ASV richness across irrigation and fertilization regimes altered the abundance of unique ASVs, cascading into shifts in dominant taxonomic-level compositions and reshaping microbial community structure (Fig 4). Redundancy Analysis (RDA) of phyla revealed strong associations between soil nutrient parameters (URE, INV, AP, and SOC) and bacterial communities across all three irrigation levels (Fig 7). Soil chemical properties, particularly SOC and AP, play a pivotal role in shaping microbial assemblages by regulating substrate and nutrient availability for microbial growth and enzymatic activity. Proteobacteria (a gram-negative bacterial phylum) are highly sensitive to environmental perturbations, including irrigation regimes [30]. Our results align with Dennis et al. [53], who showed that increasing irrigation water—specifically from slight deficit levels (e.g., I2) to higher inputs—significantly elevates Proteobacteria relative abundance. Notably, in our study, slight deficit irrigation (I2) was sufficient to sustain elevated microbial abundances, indicating that optimized water management can support microbial diversity without excessive water use. These findings contrast with Peralta et al. [42], who reported negative impacts of both excessive irrigation (I1) and moderate deficit irrigation (I3) on soil bacterial populations. This supports hypothesis H2. In our experiment, I1 (conventional irrigation) reduced microbial diversity due to waterlogging, while I3 (moderate deficit) decreased microbial abundance compared to I2. This underscores that both extreme wetting and drying negatively affect greenhouse tomato bacterial communities. The strong correlations between soil nutrients (e.g., AP, SOC) and microbial diversity highlight that integrated management of soil fertility and irrigation strategies can foster favorable conditions for sustaining robust microbial communities and enhancing soil health.

### 4.9 Correlation between soil properties, microbes, and enzyme activity

Numerous studies have demonstrated the impacts of fertilization and irrigation regimes on soil microbial communities and enzymatic activity [41,47,54]. Our research revealed positive correlations between most bacterial phyla and soil enzymes or physicochemical properties (p < 0.05 for Proteobacteria, Gemmatimonadota, and Methylomirabilota), with the exception of phosphatase (PHO). Specifically, Proteobacteria showed a strong positive correlation with urease (URE, p < 0.01), while Actinobacteriota were significantly positively associated with soil available phosphorus (AP, p < 0.05) (Fig 8). These findings highlight direct interactions between bacterial phyla and soil enzyme activity under varying irrigation and fertilization patterns. Conversely, soil pH was negatively correlated with Bacteroidota, and soil available potassium (AK) with Desulfobacterota. The current study shows that Actinobacteriota and Proteobacteria are directly linked to soil enzymes and nutrient concentrations that respond positively to irrigation and fertilization management [55]. These correlations suggest that optimizing irrigation and fertilization practices can modulate soil microbial communities and enzymatic activities, thereby influencing soil nutrient cycling processes. Such management strategies may enhance soil health, improve nutrient use efficiency, and support sustainable agricultural systems over time. Additionally, promoting beneficial microbial communities through tailored management could contribute to disease suppression, as diverse and active microbiomes inhibit soil-borne pathogens via competition and antagonism.

### 4.10 Ecological implications and long-term impacts

This study’s findings have ecological implications beyond greenhouse tomato cultivation. Irrigation and fertilization impacts on soil microbial communities and enzymatic activities may affect long-term nutrient cycling, soil fertility, and ecosystem functioning. By enhancing soil enzyme activities and promoting microbial diversity—particularly through organic fertilization and optimized irrigation practices—this study demonstrates the feasibility of improving nutrient bioavailability and soil structure, thereby fostering more sustainable agricultural systems. Moreover, elevated microbial diversity and activity can enhance disease suppression by inhibiting soil-borne pathogens through mechanisms such as resource competition, antimicrobial compound production, and induction of plant systemic resistance [56]. These outcomes could reduce dependence on chemical pesticides and promote robust crop health. In the era of climate change and the pressing need for sustainable agriculture, understanding the interplay between irrigation, fertilization, and soil microbiomes is critical. Practices that enhance soil microbial health can enhance soil carbon sequestration, mitigate greenhouse gas emissions, and strengthen the resilience of agricultural ecosystems to environmental stresses [57]. Thus, the insights from this study provide a foundation for developing management strategies that not only boost crop productivity but also advance environmental sustainability and ecosystem service provision.

### 4.11 Practical implications

The findings of this study provide actionable insights for greenhouse tomato producers seeking to optimize water and fertilizer inputs while sustaining high crop yields and enhancing soil health. Implementing slight deficit irrigation (I2)—which reduces water use by 20–30% compared to conventional irrigation (I1)—can mitigate water scarcity in arid regions without compromising yields. When combined with integrated fertilization strategies, particularly C1 (soluble organic-chemical fertilizer blends) and C4 (sole organic fertilizers), this approach enhances soil microbial activity, nutrient cycling, and long-term soil fertility. Greenhouse managers are advised to incorporate organic amendments (e.g., sheep manure) to balance nutrient supply, improve uptake efficiency, and mitigate environmental risks associated with excessive chemical fertilizer use. This supports hypothesis H4. Leveraging real-time soil moisture monitoring technologies, such as the FDS-100 soil moisture sensor used in this study, enables precise irrigation scheduling, optimizing water use efficiency and enhancing plant resilience to water stress. Appropriate irrigation-fertilization practices that elevate enzyme activity (e.g., catalase, urease) foster improved soil structure and nutrient bioavailability, ensuring sustained high yields across multiple growing seasons. Additionally, these practices promote natural disease suppression by strengthening microbial communities, reducing reliance on chemical pesticides. From an environmental sustainability perspective, optimizing water and fertilizer management in greenhouses minimizes water extraction and chemical fertilizer dependency, thereby reducing the carbon footprint. Cultivating healthy soil microbiomes also supports critical ecosystem services, including carbon sequestration and soil erosion mitigation, which are essential for long-term agricultural resilience. In conclusion, integrating moderate deficit irrigation with organic-chemical fertilizer combinations represents a robust strategy to enhance productivity, conserve resources, and foster resilient, sustainable greenhouse systems amid escalating environmental challenges.

## 5. Conclusion

This study examines fertilization and irrigation effects on bacterial community diversity/composition and their impacts on soil enzymes and physicochemical properties in greenhouse tomato soils.

(1) Reduced irrigation led to declines in bacterial richness, diversity, and soil enzyme activity. However, the application of C1 (soluble organic fertilizer combined with chemical fertilizer) mitigated these negative effects by significantly enhancing soil bacterial community diversity and ASV richness. This finding highlights the potential of integrating organic and chemical fertilizers to maintain soil health under water-limited conditions.
(2) Deficit irrigation increased soil pH, available phosphorus (AP), and nitrate nitrogen (SNO₃ ⁻ -N). Soil enzyme activity exhibited significant correlations with pH, soil organic carbon (SOC), and soil nitrate nitrogen (SNO₃ ⁻ -N), underscoring the close link between soil enzyme dynamics and key soil properties such as pH and nutrient content.
(3) Fertilization patterns also significantly influenced soil enzyme activities. Specifically, treatments incorporating organic fertilizers (e.g., C1 and C4) enhanced the activity of key soil enzymes (catalase, urease, and invertase), whereas soluble chemical fertilizer treatments (C2) reduced enzyme activity. This demonstrates that organic fertilizers play a critical role in promoting soil enzyme activity and health, thereby enhancing microbial function and nutrient cycling. These results emphasize the need to integrate organic fertilizers into conventional practices to improve soil health and ecosystem sustainability.

Novel contributions of this study include the identification of specific irrigation-fertilization combinations (I2-C1 and I2-C4) that optimize microbial diversity and soil enzyme activity, providing a balanced approach to enhance water-use efficiency and nutrient management in greenhouses. The study also clarifies how irrigation and fertilization regimes influence the abundance of dominant phyla (Actinobacteriota, Proteobacteria, Chloroflexi, and Acidobacteriota), thereby shaping bacterial composition, richness, and community structure in greenhouse tomato soils.

These findings can guide the development of irrigation and fertilization strategies to sustainably enhance soil fertility, bacterial structure, and community health while mitigating environmental pollution risks in greenhouse tomato production systems in China.

## Supporting information

S1 Table Effective sequence statistics table. (CSV)

S2 Table Alpha diversity index table. (XLSX)

S3 Table ASV species taxonomic information sheet. (CSV)

## References

[1] Li X, Hu X, Song S, Sun D. Greenhouse Management for Better Vegetable Quality, Higher Nutrient Use Efficiency, and Healthier Soil. Horticulturae. 2022;8(12):1192. doi: 10.3390/horticulturae8121192

[2] Liu J, Zhang J, Shi Q, Liu X, Yang Z, Han P, et al. The Interactive Effects of Deficit Irrigation and Bacillus pumilus Inoculation on Growth and Physiology of Tomato Plant. Plants (Basel). 2023;12(3):670. doi: 10.3390/plants12030670 36771756 PMC9919795

[3] Wang X, Yun J, Shi P, Li Z, Li P, Xing Y. Root growth, fruit yield and water use efficiency of greenhouse grown tomato under different irrigation regimes and nitrogen levels. J Plant Growth Regul. 2018;38(2):400–15. doi: 10.1007/s00344-018-9850-7

[4] Zotarelli L, Dukes MD, Scholberg JMS, Muñoz-Carpena R, Icerman J. Tomato nitrogen accumulation and fertilizer use efficiency on a sandy soil, as affected by nitrogen rate and irrigation scheduling. Agricultural Water Management. 2009;96(8):1247–58. doi: 10.1016/j.agwat.2009.03.019

[5] Geldhof B, Pattyn J, Van de Poel B. From a different angle: genetic diversity underlies differentiation of waterlogging-induced epinasty in tomato. Front Plant Sci. 2023;14:1178778. doi: 10.3389/fpls.2023.1178778 37324684 PMC10264670

[6] Ministry of Water Resources of the People’s Republic of China. 2019 Annual Bulletin of China’s Water Resources. Ministry of Water Resources of the People’s Republic of China. 2020.

[7] Gronau S, Winter E, Grote U. Papyrus, forest resources and rural livelihoods: A village computable general equilibrium analysis from northern Zambia. Natural Resources. 2018;9:268–96.

[8] Qu Z, Qi X, Liu Y, Liu K, Li C. Interactive effect of irrigation and polymer-coated potassium chloride on tomato production in a greenhouse. Agricultural Water Management. 2020;235:106149. doi: 10.1016/j.agwat.2020.106149

[9] Mukherjee S, Dash PK, Das D, Das S. Growth, Yield and Water Productivity of Tomato as Influenced by Deficit Irrigation Water Management. Environ Process. 2023;10(1). doi: 10.1007/s40710-023-00624-z

[10] Alzoheiry AM, Ghumaiz NSA, Motawei MI, Kassem MA. Water productivity and growth parameters of Fawn-tall fescue and Tekapo-orchard grass under deficit irrigation in arid zones. Braz J Biol. 2023;84:e272544. doi: 10.1590/1519-6984.272544 37222377

[11] Wu Y, Yan S, Fan J, Zhang F, Xiang Y, Zheng J, et al. Responses of growth, fruit yield, quality and water productivity of greenhouse tomato to deficit drip irrigation. Scientia Horticulturae. 2021;275:109710. doi: 10.1016/j.scienta.2020.109710

[12] Kai T, Tamaki M. Effect of Organic and Chemical Fertilizer Application on Growth, Yield, and Soil Biochemical Properties of Landrace <;i>;Brassica napus<;/i>; L. Leaf-and-Stem Vegetable and Landrace (Norabona). JACEN. 2020;09(04):314–30. doi: 10.4236/jacen.2020.94023

[13] Lv H, Lin S, Wang Y, Lian X, Zhao Y, Li Y, et al. Drip fertigation significantly reduces nitrogen leaching in solar greenhouse vegetable production system. Environ Pollut. 2019;245:694–701. doi: 10.1016/j.envpol.2018.11.042 30500748

[14] Dan X, Meng L, He M, Chen S, He X, Zhao C, et al. Gross N transformations and plant N use efficiency in intensive vegetable production soils. Soil Biology and Biochemistry. 2022;174:108817. doi: 10.1016/j.soilbio.2022.108817

[15] Alhassan I, Maiga A. Artificial Intelligence in Climate Change Mitigation: A Socio-Technical Framework for Evaluating Implementation Effectiveness and Systemic Impact. VP. 2025;11(01):171–90. doi: 10.4236/vp.2025.111014

[16] Norse D, Ju X. Environmental costs of China’s food security. Agriculture, Ecosystems & Environment. 2015;209:5–14. doi: 10.1016/j.agee.2015.02.014

[17] Wu Y, Yan S, Fan J, Zhang F, Zhao W, Zheng J, et al. Combined effects of irrigation level and fertilization practice on yield, economic benefit and water-nitrogen use efficiency of drip-irrigated greenhouse tomato. Agricultural Water Management. 2022;262:107401. doi: 10.1016/j.agwat.2021.107401

[18] Susan I, Deodatus K, Cargele M. Maize response to chemical and microbial products on two Tanzanian soils. CCSE. 2019.

[19] Wang L, Hamel C, Lu P, Wang J, Sun D, Wang Y, et al. Using enzyme activities as an indicator of soil fertility in grassland - an academic dilemma. Front Plant Sci. 2023;14:1175946. doi: 10.3389/fpls.2023.1175946 37484467 PMC10360189

[20] Leifeld J, Franko U, Schulz E. Thermal stability responses of soil organic matter to long-term fertilization practices. Biogeosciences. 2006;3(3):371–4. doi: 10.5194/bg-3-371-2006

[21] Megyes M, Borsodi AK, Árendás T, Márialigeti K. Variations in the diversity of soil bacterial and archaeal communities in response to different long-term fertilization regimes in maize fields. Applied Soil Ecology. 2021;168:104120. doi: 10.1016/j.apsoil.2021.104120

[22] Fierer N, Lauber CL, Ramirez KS, Zaneveld J, Bradford MA, Knight R. Comparative metagenomic, phylogenetic and physiological analyses of soil microbial communities across nitrogen gradients. ISME J. 2012;6(5):1007–17. doi: 10.1038/ismej.2011.159 22134642 PMC3329107

[23] Coolon JD, Jones KL, Todd TC, Blair JM, Herman MA. Long-term nitrogen amendment alters the diversity and assemblage of soil bacterial communities in tallgrass prairie. PLoS One. 2013;8(6):e67884. doi: 10.1371/journal.pone.0067884 23840782 PMC3695917

[24] Zhao J, Ni T, Li J, Lu Q, Fang Z, Huang Q, et al. Effects of organic–inorganic compound fertilizer with reduced chemical fertilizer application on crop yields, soil biological activity and bacterial community structure in a rice–wheat cropping system. Applied Soil Ecology. 2016;99:1–12. doi: 10.1016/j.apsoil.2015.11.006

[25] Feng Y, Chen R, Hu J, Zhao F, Wang J, Chu H, et al. Bacillus asahii comes to the fore in organic manure fertilized alkaline soils. Soil Biology and Biochemistry. 2015;81:186–94. doi: 10.1016/j.soilbio.2014.11.021

[26] Kai T, Tamaki M. Effect of Organic and Chemical Fertilizer Application on Growth, Yield, and Soil Biochemical Properties of Landrace <;i>;Brassica napus<;/i>; L. Leaf-and-Stem Vegetable and Landrace (Norabona). JACEN. 2020;09(04):314–30. doi: 10.4236/jacen.2020.94023

[27] Doemel WN, Brock TD. pH of very acid soils. Nature. 1971;229(5286):574. doi: 10.1038/229574a0 16059352

[28] Gai X, Liu H, Zhai L, Tan G, Liu J, Ren T, et al. Vegetable yields and soil biochemical properties as influenced by fertilization in Southern China. Applied Soil Ecology. 2016;107:170–81. doi: 10.1016/j.apsoil.2016.06.001

[29] Bao S. Soil Agricultural Chemistry Analysis. Agriculture Press. 1999.

[30] Schimel J, Balser TC, Wallenstein M. Microbial stress-response physiology and its implications for ecosystem function. Ecology. 2007;88(6):1386–94. doi: 10.1890/06-0219 17601131

[31] Sivapalan K, Fernando V, Thenabadu MW. Humified phenol-rich plant residues and soil urease activity. Plant Soil. 1983;70(1):143–6. doi: 10.1007/bf02374757

[32] Johnson JL, Temple KL. Some Variables Affecting the Measurement of “Catalase Activity” in Soil. Soil Science Soc of Amer J. 1964;28(2):207–9. doi: 10.2136/sssaj1964.03615995002800020024x

[33] Tabatabai MA, Bremner JM. Use of p-nitrophenyl phosphate for assay of soil phosphatase activity. Soil Biology and Biochemistry. 1969;1(4):301–7. doi: 10.1016/0038-0717(69)90012-1

[34] Zeng X-Y, Li S-W, Leng Y, Kang X-H. Structural and functional responses of bacterial and fungal communities to multiple heavy metal exposure in arid loess. Sci Total Environ. 2020;723:138081. doi: 10.1016/j.scitotenv.2020.138081 32220739

[35] Chen S, Zhou Y, Chen Y, Gu J. fastp: an ultra-fast all-in-one FASTQ preprocessor. Bioinformatics. 2018;34(17):i884–90. doi: 10.1093/bioinformatics/bty560 30423086 PMC6129281

[36] Magoč T, Salzberg SL. FLASH: fast length adjustment of short reads to improve genome assemblies. Bioinformatics. 2011;27(21):2957–63. doi: 10.1093/bioinformatics/btr507 21903629 PMC3198573

[37] Rodriguez A, Heavner B, Kesselman C, et al. Reproducible big data science: A case study in continuous FAIRness. Archive of “PLoS ONE”. 2019;18(11): e0294883. 10.1371/journal.pone.0294883 PMC10662719 37988378

[38] Callahan BJ, McMurdie PJ, Holmes SP. Exact sequence variants should replace operational taxonomic units in marker-gene data analysis. ISME J. 2017;11(12):2639–43. doi: 10.1038/ismej.2017.119 28731476 PMC5702726

[39] Chen L-F, He Z-B, Wu X-R, Du J, Zhu X, Lin P-F, et al. Linkages between soil respiration and microbial communities following afforestation of alpine grasslands in the northeastern Tibetan Plateau. Applied Soil Ecology. 2021;161:103882. doi: 10.1016/j.apsoil.2021.103882

[40] Chen H, Shang Z, Cai H, Zhu Y. Irrigation Combined with Aeration Promoted Soil Respiration through Increasing Soil Microbes, Enzymes, and Crop Growth in Tomato Fields. Catalysts. 2019;9(11):945. doi: 10.3390/catal9110945

[41] Muhammad I, Yang L, Ahmad S. Irrigation and nitrogen fertilization alter soil bacterial communities, soil enzyme activities, and nutrient availability in maize crop. Front Microbiol. 2022;13:833758. 35185852 10.3389/fmicb.2022.833758 PMC8851207

[42] Peralta AL, Ludmer S, Matthews JW, Kent AD. Bacterial community response to changes in soil redox potential along a moisture gradient in restored wetlands. Ecological Engineering. 2014;73:246–53. doi: 10.1016/j.ecoleng.2014.09.047

[43] Wu Y, Si W, Yan S, Wu L, Zhao W, Zhang J, et al. Water consumption, soil nitrate-nitrogen residue and fruit yield of drip-irrigated greenhouse tomato under various irrigation levels and fertilization practices. Agricultural Water Management. 2023;277:108092. doi: 10.1016/j.agwat.2022.108092

[44] Li P, Wei W, Lang M. Effects of water content on gross nitrogen transformation rates in forest land and grassland soils. Ying Yong Sheng Tai Xue Bao. 2022;33(1):59–66. doi: 10.13287/j.1001-9332.202201.022 35224926

[45] Pajares S, Gallardo JF, Masciandaro G, Ceccanti B, Etchevers JD. Enzyme activity as an indicator of soil quality changes in degraded cultivatedAcrisolsin the Mexican Trans‐volcanic Belt. Land Degrad Dev. 2011;22(3):373–81. doi: 10.1002/ldr.992

[46] Nadarajah K, Abdul Rahman NSN. The microbial connection to sustainable agriculture. Plants (Basel). 2023;12(12):2307. 37375932 10.3390/plants12122307 PMC10303550

[47] Yuan X, Zhang J, Chang F, Wang X, Zhang X, Luan H, et al. Effects of nitrogen reduction combined with bio-organic fertilizer on soil bacterial community diversity of red raspberry orchard. PLoS One. 2023;18(7):e0283718. doi: 10.1371/journal.pone.0283718 37432967 PMC10335678

[48] Sun Z, Lin M, Du C, Hao Y, Zhang Y, Wang Z. The use of manure shifts the response of α-diversity and network while not β-diversity of soil microbes to altered irrigation regimes. Applied Soil Ecology. 2022;174:104423. doi: 10.1016/j.apsoil.2022.104423

[49] Li H, Wang H, Jia B, Li D, Fang Q, Li R. Irrigation has a higher impact on soil bacterial abundance, diversity and composition than nitrogen fertilization. Sci Rep. 2021;11(1):16901. doi: 10.1038/s41598-021-96234-6 34413369 PMC8377015

[50] O AA, A IA, S UA. Influence of combined application of P fertilizer and lime on Mucuna flagellipes nodulation, growth and yield. African Journal of Plant Science. 2019;13(3):47–58.

[51] He J, He Y, Gao W, Chen Y, Ma G, Ji R, et al. Soil depth and agricultural irrigation activities drive variation in microbial abundance and nitrogen cycling. CATENA. 2022;219:106596. doi: 10.1016/j.catena.2022.106596

[52] Zhang X, Li J, Shao L, Qin F, Yang J, Gu H, et al. Effects of organic fertilizers on yield, soil physico-chemical property, soil microbial community diversity and structure of Brassica rapa var. Chinensis. Front Microbiol. 2023;14:1132853. doi: 10.3389/fmicb.2023.1132853 37323918 PMC10266463

[53] Dennis PG, Miller AJ, Hirsch PR. Are root exudates more important than other sources of rhizodeposits in structuring rhizosphere bacterial communities? FEMS Microbiol Ecol. 2010;72(3):313–27. doi: 10.1111/j.1574-6941.2010.00860.x 20370828

[54] Gu S, Hu Q, Cheng Y, Bai L, Liu Z, Xiao W, et al. Application of organic fertilizer improves microbial community diversity and alters microbial network structure in tea (Camellia sinensis) plantation soils. Soil and Tillage Research. 2019;195:104356. doi: 10.1016/j.still.2019.104356

[55] Ming A, Yang Y, Liu S, Wang H, Li Y, Li H, et al. Effects of Near Natural Forest Management on Soil Greenhouse Gas Flux in Pinus massoniana (Lamb.) and Cunninghamia lanceolata (Lamb.) Hook. Plantations. Forests. 2018;9(5):229. doi: 10.3390/f9050229

[56] Mendes R, Garbeva P, Raaijmakers JM. The rhizosphere microbiome: significance of plant beneficial, plant pathogenic, and human pathogenic microorganisms. FEMS Microbiol Rev. 2013;37(5):634–63. doi: 10.1111/1574-6976.12028 23790204

[57] Singh S, P A, Ahlawat U, Changdeo WB, Rehsawla R, Naruka A, et al. Mechanisms and Applications of Microbial Biotechnology in Soil Health and Agricultural Productivity: A Review. J Adv Biol Biotechnol. 2024;27(7):1420–38. doi: 10.9734/jabb/2024/v27i71104

